# Procoagulant factors and future risk of arterial cardiovascular disease in patients with prior venous thrombosis: A cohort study

**DOI:** 10.1002/jha2.618

**Published:** 2023-01-17

**Authors:** Eng Soo Yap, Willem M. Lijfering, Jasmijn F. Timp, Frits R. Rosendaal, Suzanne C. Cannegieter, Luuk J. J. Scheres

**Affiliations:** ^1^ Department of Clinical Epidemiology Leiden University Medical Center Leiden The Netherlands; ^2^ Department of Laboratory Medicine National University Hospital Singapore Singapore; ^3^ Kennisinstituut, Federatie Medisch Specialisten Utrecht The Netherlands; ^4^ Einthoven Laboratory for Experimental Vascular Medicine Leiden University Medical Center Leiden The Netherlands; ^5^ Department of Internal Medicine Section of Thrombosis and Haemostasias Leiden University Medical Center Leiden The Netherlands; ^6^ Department of Internal Medicine Radboud University Medical Center Nijmegen The Netherlands

**Keywords:** cardiovascular disease, coagulation factors, epidemiology, risk factors, venous thrombosis

## Abstract

Patients with venous thrombosis (VT) are at increased risk of future arterial cardiovascular disease (CVD) (i.e., myocardial infarction, ischemic stroke or peripheral artery disease). We investigated whether shared risk factors for VT and CVD are associated with the levels of procoagulant factors (fibrinogen, factor VIII, and von Willebrand factor), and whether the relationship between these risk factors and subsequent CVD was mediated through these procoagulant factors in patients with VT. In a follow‐up study consisting of 4956 patients with VT, 2176 patients (44%) provided blood samples and were linked to the Dutch Hospital registry of Statistics Netherlands to identify hospital admissions or procedures for subsequent CVD. In total, 52 CVD events occurred over a follow‐up of 11,124 years, with an incidence rate of 4.7 per 1000 patient years (95% confidence intervals 3.5–6.1). Increasing age, male sex, smoking history, major illnesses, dyslipidemia, and impaired fasting glucose levels were associated with increased CVD risk. Procoagulant factor levels were also associated with CVD risk. When adjusted for these procoagulant factors, the association between the risk factors and CVD attenuated partially. This study provides evidence that procoagulant factors can partially explain the association between increased risks of subsequent CVD in patients with previous VT.

## 1 INTRODUCTION

The relationship between venous thrombosis (VT) and subsequent arterial cardiovascular disease (CVD) is thought to be due to the effects of shared risk factors (i.e., factors that increase both VT and CVD risk) such as older age, male sex, body mass index (BMI) ≥25 kg/m^2^, and lifestyle‐related factors [1, 2, 3, 4]. This hypothesis was previously confirmed in the Multiple Environmental and Genetic Assessment of risk factors (MEGA) follow‐up study, where the crude relative risk of CVD after VT (compared with controls without a recent history of VT) decreased from 2.2 (95% confidence intervals [CI] 1.2–3.8) to 1.3 (95% CI 0.7–2.5) when abovementioned shared risk factors were taken into account [5]. Nevertheless, although these shared risk factors explain an increased risk of subsequent CVD in patients with previous VT, the underlying pathophysiological mechanism remains unknown. Since increased levels of the procoagulant factors fibrinogen, factor VIII (FVIII) and von Willebrand factor (vWF) are associated with an increased risk of both VT and CVD and also with shared risk factors such as increasing age and obesity[6, 7, 8, 9, 10, 11, 12, 13], we hypothesize that increased levels of coagulation factors might, at least in part, explain the increased risk of CVD in patients with prior VT.

We therefore investigated whether the relationship between shared risk factors and subsequent CVD risk could be explained through these procoagulant factors in a population of patients with previous VT.

## 2 METHODS

### 2.1 Study design

Details of the MEGA study have been described previously [14]. Briefly, between March 1999 and September 2004, consecutive patients aged 18–70 years with a first deep vein thrombosis and/or pulmonary embolism were included in the MEGA study from six anticoagulation clinics in the Netherlands. The MEGA study was approved by the medical ethics committee of the Leiden University Medical Center, the Netherlands. Written informed consent was obtained from the study participants.

### 2.2 Data collection

MEGA participants were asked to complete a questionnaire on risk factors for both VT and CVD. The index date was defined as the date of VT diagnosis.

Patients who had discontinued anticoagulation therapy for approximately 3 months or were on anticoagulant therapy for over 1 year visited the anticoagulation clinic for an interview and were asked to provide a blood sample [15]. For logistical reasons, blood sampling was requested until June 2002. The blood samples were used to assess levels of the coagulation factors fibrinogen, FVIII, vWF, creatinine (to estimate the glomerular filtration rate [eGFR in ml/min]), and lipid levels, including total cholesterol, high‐density lipoprotein (HDL)‐cholesterol and triglycerides. A more detailed description of the coagulation factor assays and the measurement of the eGFR can be found elsewhere [16, 17, 18]. Total cholesterol, HDL, and triglyceride concentrations were measured on previously unthawed fasting serum samples stored at −80°C. To determine the low‐density lipoprotein (LDL)‐cholesterol concentration, the Friedewald formula was used [19].

### 2.3 Follow‐up

The vital status of all MEGA participants was retrieved from the Dutch population registry between February 2007 and May 2009, as described previously [14]. To identify individuals with a CVD event, participants of the MEGA study were linked to the Dutch Hospital Data registry as described previously [5]. From 1995 onward, the Dutch Hospital Data registry provides nationwide coverage of data on all hospital admissions and in‐hospital procedures. MEGA study participants were linked to this registry through sex, date of birth and postal code. Of the 4956 patients in the MEGA study with a VT, 4539 (92%) could be uniquely linked to the registry. After this linkage, the patients with a CVD event (e.g., the study outcome of interest) were identified, which was a composite of both hospital admission for a CVD diagnosis or a CVD‐related procedure. The diagnoses corresponding with the hospital admissions are encoded according to the International Classification of Diseases, ninth revision, clinical modification (International Classification of Disease (ICD)‐9‐CM). Hospital admission for acute CVD was defined as myocardial infarction (ICD‐9‐CM codes 4100–4109, ICD‐10‐CM code I21), ischemic stroke (ICD‐9‐CM codes 433, 4330–4333, 4338, 4339, 434, 4340, 4341 and 4349, ICD‐10‐CM code I63, I64), or peripheral vascular disease (ICD‐9‐CM codes 4439, ICD‐10‐CM code I73.9). We chose these specific events (myocardial infarction, ischemic stroke and symptomatic peripheral vascular disease) as outcomes (opposed to for example transient ischemic attacks or angina) since these events would certainly be captured by the Dutch Hospital Data registry as they require hospitalization due to the severity of the disease. For the CVD‐related procedures, we considered procedures that are performed for symptomatic CVD: coronary bypass (5361, 53610, 53611, 53612), percutaneous transluminal (coronary) angioplasty (88370, 88372, 88373, 88374, 88378, 883600, 883606, 883607, 883609, 883656), or selective arterial thrombolysis of the extremities (88362, 883627, 883628).

Patients with CVD between 1995 and the index date, and patients for whom either the date of inclusion in the study or the date of diagnosis was unknown, were excluded.

### 2.4 Statistical analysis

Observation time started at the time of blood draw and ended on the date of either a first CVD event, VT recurrence, death or date on which the vital status was retrieved (between February 2007 and May 2009), whichever came first. Events (either diagnoses or procedures) of myocardial infarction, ischemic stroke, and peripheral vascular disease (deaths and first events) were analyzed as a combined endpoint. The incidence rate with 95% CIs (based on a Poisson distribution) of CVD was calculated by dividing the number of cardiovascular events by the sum of the observation time.

The definitions for raised total cholesterol (>6.2 mmol/L), low HDL (≤1.55 mmol/L), raised TG (>2.26 mmol/L), and raised LDL (≥4.9 mmol/L) are based on both American and European guideline [20, 21]. The definitions for impaired fasting glucose (fasting blood glucose level between 5.6 and 6.9 mmol/L) and diabetes mellitus (fasting blood glucose level ≥7 mmol/L) are based on World Health Organization guidelines [22].

**FIGURE 1:**
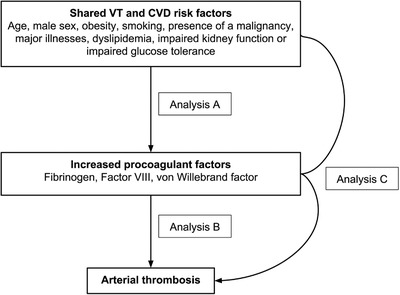
Diagram of the investigated associations between venous thrombosis (VT) and cardiovascular disease (CVD) risk factors. The association between the shared risk factors and procoagulant factors (analysis A), between procoagulant factors and risk of arterial thrombosis (analysis B) and the potential mediating effect of procoagulant factors in the risk of these risk factors on CVD risk (analysis C) are explored.

The studied associations are shown in Figure 1. We first assessed whether the shared risk factors (increasing age, male sex, BMI ≥ 25 kg/m^2^, smoking, presence of malignancy, major illnesses, dyslipidemia, impaired kidney function, or diabetes mellitus) were associated with changes in the procoagulant factors levels (Analysis A). We used linear regression to estimate the mean increase in levels of fibrinogen, FVIII and vWF for the presence of a particular risk factor. Additionally, we adjusted for age and sex to adjust for their effects on changes in these levels. Secondly, we investigated whether changes in levels of procoagulant factors were associated with increased risk of CVD events (Analysis B). Fibrinogen, FVIII and vWF were a priori categorized into six categories based on levels in the MEGA random digit dialing control population (<25th percentile [reference], 25–50th percentile, 50–75th percentile, 75–90th percentile, 90–97.5th percentile and >97.5th percentile). The relative risk of CVD events expressed as hazard ratios (HR) with 95% CI was compared with the lowest 25th percentile of all three procoagulant factors as the reference group and adjusted for age, sex and the use of vitamin K antagonists; the latter as a time dependent variable, as its use could vary over time [23]. Finally, we determined if shared risk factors were associated with an increased risk for CVD events using multivariate Cox regression analysis to estimate HRs with 95% CIs (Analysis C). In addition, for each individual risk factor, we adjusted for fibrinogen, FVIII, and vWF levels to explore if any increased risk for CVD events was mediated through these coagulation factors (mediation analysis). Statistical analyses were performed with SPSS for Windows, release 22.0 (SPSS Inc, Chicago, Ill).

## 3 RESULTS

### 3.1 General characteristics

In total, 4956 patients with VT were included in the MEGA study (Figure S1 is a flow chart of the selection of the study population). Of these, 417 patients (8.4%) could not be linked to the Dutch Hospital Data registry. Of the 4539 patients remaining, 58 were excluded as they had a CVD event before the index date. Of these 4481 patients, 2176 (49%) patients provided a blood sample (which was requested until Jun 2002) and were therefore available for the analysis. Table 1 shows the baseline characteristics and the distribution of the risk factors for the study population. Of all patients combined (with and without blood sample), 2039 (46%) were men and the mean age at enrollment was 49 years (interquartile range [IQR] 25–75th percentile: 39–60 years). Of the patients who provided blood samples, 990 (46%) were men, and the mean age was 48 years (IQR: 38–58). The patients who provided blood had a malignancy less often than those who did not provide a blood sample but had otherwise similar clinical characteristics as compared with the entire cohort. In the patients with blood samples available, 99 patients had CVD or venous event before the blood draw and follow‐up time of 15 patients cannot be determined, a final 2062 patients with VT were included in the further analyses (Figure S2). In these, the mean follow‐up time was 5.9 years (IQR 4.9–6.8 years) with 131 (6.4%) deaths occurring during follow‐up. In total, 52 CVD events occurred over a follow‐up time of 11,124 patient years, at an incidence rate of 4.7 per 1000 patient years (95% CI 3.5–6.1).

**TABLE 1:** General characteristics of cases with venous thrombosis from the MEGA study

	**All cases (*n* = 4481)**	**Cases with blood samples (*n* = 2176)**
General characteristics		
Male sex, *n* (%)	2039 (46)	990 (46)
Age, mean (IQR)	49 (39–60)	48 (38–58)
BMI, mean (IQR)	26.8 (23.7–29.2)	26.9 (23.8–29.2)
BMI ≥ 25, *n* (%)	2485 (63)	1301 (63)
Malignancy <5 years, *n* (%)	420 (9.4)	113 (5.2)
Non‐smoker, *n* (%)	1413 (35)	742 (35)
Major illnesses, *n* (%)	222 (5)	97 (5)
Blood parameters		
Total Cholesterol (mmol/L), mean (IQR)	NA	5.6 (4.9–6.3)
HDL (mmol/L), mean (IQR)	NA	1.3 (1.0–1.5)
Triglycerides (mmol/L), mean (IQR)	NA	1.6 (1.0–1.9)
LDL (mmol/L), mean (IQR)	NA	4 (3.2–4.7)
eGFR (ml/min), mean (IQR)	NA	113 (92–132)
Fasting glucose (mmol/L), mean (IQR)	NA	5.1 (4.4–5.3)
Fibrinogen (g/L), mean (IQR)	NA	3.5 (3.0–3.9)
Factor VIII (IU/dl), mean (IQR)	NA	141 (108–166)
von Willebrand Factor (IU/dl), mean (IQR)	NA	150 (110–176)

Abbreviations: BMI, body mass index; eGFR, estimated glomerular filtration rate; HDL, high‐density lipoprotein; LDL, low‐density lipoprotein; NA, not available.

To assess whether the relationship between shared risk factors and subsequent CVD risk could be explained through procoagulant factors in patients with previous VT, we did three analyses.

### 3.2 Analysis 1. Association between shared risk factors and procoagulant factor levels

Table 2 shows that the studied risk factors (i.e., increasing age, BMI ≥ 25 kg/m^2^ or presence of a malignancy, major illness, dyslipidemia, impaired fasting glucose levels, and impaired kidney function) were associated with higher levels of fibrinogen, FVIII and vWF. Increasing age, impaired glucose levels, and presence of major illnesses, malignancy or impaired kidney function were associated with the highest mean difference in procoagulant factor levels.

**TABLE 2:** Difference in procoagulant factor levels for common cardiovascular risk factors in 2062 patients with venous thrombosis

			Mean difference (95%CI)			
	Fibrinogen (g/L)	Fibrinogen (g/L)*	FVIII (IU/dl)	FVIII (IU/dl)*	vWF (IU/dl)	vWF (IU/dl)*
Age (years)						
40–50 versus <40	0 (−0.09–0.09)	0.03 (−0.60–−0.12)	7 (1–12)	7 (2–13)	9 (3–16)	10 (3–16)
50–60 versus <40	0.18 (0.09–0.26)	0.24 (0.15–0.33)	11 (6–16)	11 (6–16)	23 (15–29)	21 (14–28)
>60 versus <40	0.38 (0.28–0.48)	0.47 (0.37–0.58)	25 (19–31)	27 (20–33)	43 (36–51)	43 (35–51)
Sex						
Men versus women	−0.14 (−0.20–−0.07)	−0.24 (−0.31–0.17)	2 (−2–6)	−3 (−8–1)	10 (5–16)	2 (−4–7)
BMI (kg/m2)						
≥25 versus <25	0.20 (0.13–0.27)	0.20 (0.13–0.27)	6 (1–10)	4 (0–8)	7 (1–13)	4 (−1–10)
Malignancy <5 years						
Yes versus no	0.31 (0.16–0.46)	0.20 (0.06–0.35)	16 (7–25)	10 (1–19)	29 (17–41)	19 (7–31)
Smoking						
Ever versus never	0.03 (−0.05–0.10)	0.03 (−0.04–0.10)	−4 (−9–0)	−6 (−10–−1)	9 (3–15)	6 (0–11)
Major illness						
Ever versus never	0.31 (0.15–0.48)	0.23 (0.07–0.39)	19 (9–29)	15 (5–25)	34 (21–47)	29 (16–41)
Total cholesterol (mmol/L)						
>6.2 versus ≤6.2	0.11 (0.04–0.19)	0.05 (−0.02–0.13)	11 (6–15)	7 (2–12)	13 (7–19)	7 (1–13)
HDL (mmol/L)						
≤1.55 versus >1.55	0.08 (0.01–0.16)	0.18 (0.10–0.26)	3 (−2–7)	4 (−1–9)	9 (3–15)	10 (3–16)
Triglycerides (mmol/L)						
>2.26 versus ≤2.26	0.20 (0.11–0.30)	0.23 (0.14–0.32)	16 (10–21)	15 (10–21)	24 (17–32)	22 (15–29)
LDL (mmol/L)						
≥4.9 versus <4.9	0.16 (0.07–0.24)	0.13 (0.04–0.21)	10 (5–16)	8 (2–13)	16 (9–23)	10 (3–17)
Kidney function, eGFR (ml/min)						
<60 versus ≥60	0.70 (0.48–0.91)	0.53 (0.31–0.74)	44 (30–57)	35 (22–48)	64 (47–82)	50 (33–68)
Fasting glucose (mmol/L)						
5.6‐<7 versus <5.6	0.15 (0.05–0.25)	0.11 (0.01–0.21)	14 (8–20)	10 (4–16)	18 (10–26)	10 (1–18)
≥7 versus <5.6	0.26 (0.10–0.42)	0.19 (0.03–0.35)	32 (22–42)	27 (17–37)	28 (15–41)	19 (6–31)

* Adjusted for age and/or sex, where appropriate

FVIII, factor VIII. vWF, von Willebrand factor. BMI, body mass index. HDL High‐density lipoprotein. LDL, Low‐density lipoprotein.

eGFR, estimated glomular filtration rate. 95%CI, 95% confidence intervals.

### 3.3 Analysis 2. Association between procoagulant factor levels and risk of arterial cardiovascular events

Table 3 shows a stepwise increase in risk of CVD events for increasing percentiles of the studied procoagulant factors **with the highest and steepest risk increase seen in >90th percentiles**. With the ≤25th percentile of coagulation factors as a reference, the highest HRs for CVD events were observed in the 97.5th percentile: HR 8.8 (95% CI 2.8–27.6) for fibrinogen, HR 2.4 (95% CI 0.9–6.7) for FVIII and HR 5.8 (95% CI 1.3–25.7) for vWF. After adjustments for age, sex, and use of vitamin K antagonists, these HRs were 5.4 (95% CI 1.7–17.7) for fibrinogen, 1.5 (95% CI 0.5–4.1) for FVIII and 2.7 (95% CI 0.6–12.4) for vWF.

**TABLE 3:** Risk of arterial cardiovascular disease by percentiles of the studied coagulation factors

	**Observation years**	**cases (n)**	**CVD (n)**	**Incidence rate per 1000 PY (95%CI)**	**HR (95%CI)**	**HR (95%CI)** *
Fibrinogen level (g/L)						
≤25 th percentile (≤2.85)	2233	404	5	2.2 (0.8–5.0)	1.0 (ref)	1.0 (ref)
25th–50th percentile (2.85‐3.26)	2561	469	9	3.5 (1.7–6.4)	1.6 (0.5–4.7)	1.3 (0.4–3.8)
50th–75th percentile (3.26‐3.69)	2680	491	9	3.4 (1.6–6.2)	1.5 (0.5–4.5)	1.2 (0.4–3.7)
75th–90th percentile (3.69‐4.21)	2061	383	13	6.3 (3.5–10.5)	2.8 (1.0–7.9)	2.0 (0.7–5.7)
90th–97.5th percentile (4.21‐5.02)	1234	230	9	7.3 (3.6–13.4)	3.3 (1.1–9.8)	2.9 (1.0–−8.8)
>95th percentile (>5.02)	357	85	7	19.6 (8.6–38.8)	8.8 (2.8–27.6)	5.4 (1.7–17.7)
Factor VIII level (IU/dl)						
≤25 th percentile (≤89)	1320	226	6	4.5 (1.8–9.5)	1.0 (ref)	1.0 (ref)
25th–50th percentile (89–109)	1799	314	7	3.9 (1.7–7.7)	0.9 (0.3–2.5)	0.8 (0.3–2.4)
50th–75th percentile (109–135)	2767	501	7	2.5 (1.1–5.0)	0.6 (0.2–1.6)	0.4 (0.1–1.3)
75th–90th percentile (135–166)	2766	523	11	4.0 (2.1–6.9)	0.9 (0.4–2.4)	0.6 (0.2–1.6)
90th–97.5th percentile (166–199)	1573	301	11	7.0 (3.7–12.2)	1.5 (0.6–2.4)	1.0 (0.4–2.7)
>97.5th percentile (>199)	899	197	10	11.1 (19.8–19.8)	2.4 (0.9–6.7)	1.5 (0.5–4.1)
von Willebrand factor level (IU/dl)						
≤25 th percentile (≤81)	934	157	2	2.1 (0.4–7.1)	1.0 (ref)	1.0 (ref)
25th–50th percentile (81–103)	1563	280	7	4.5 (2.0–8.9)	2.1 (0.4–10.0)	2.0 (0.4–9.6)
50th–75th percentile (103–128)	2346	426	4	1.7 (0.5–4.1)	0.8 (0.1–4.4)	0.7 (0.1–3.6)
75th–90th percentile (128–164)	3238	593	13	4.0 (2.2–6.7)	1.9 (0.4–8.3)	1.3 (0.3–5.7)
90th–97.5th percentile (164–210)	1998	374	13	6.5 (3.6–10.9)	3.0 (0.7–13.5)	1.7 (0.4–7.6)
>97.5th percentile (>210)	1046	232	13	12.4 (6.9–20.7)	5.8 (1.3–25.7)	2.7 (0.6–12.4)

Abbreviations: CVD, cardiovascular events; PY, person years; HR, hazard ratio; 95%CI, 95% confidence intervals.

* Adjusted for age, sex, and use of vitamin K antagonists.

### 3.4 Analysis 3a. Association between shared risk factors and arterial cardiovascular events

Most shared risk factors (increasing age, male sex, smoking history, major illness, raised total cholesterol, raised TG, raised LDL, impaired fasting glucose levels) were associated with an increased risk of a CVD event, as shown in Table 4. Potential exceptions were, BMI ≥ 25 kg/m^2^, HR 1.5 (95% CI 0.8–3.0) as compared with normal weight, HDL ≤ 1.55 mmol/L compared with HDL > 1.55 mmol/L HR 1.6 (95% CI 0.8–3.3) and an eGFR <60 ml/min compared with and eGFR ≥ 60 ml/min, HR 3.0 (0.9–9.7), although point estimates still showed an (as expected) increase in risk of CVD events in patients who shared these risk factors.

**TABLE 4:** Risk of arterial cardiovascular events for known cardiovascular risk factors

	Fibrinogen	FVI II	vWF	Obs yrs	cases (n)**	CVD (n)	IR (95% CI)	HR (95% CI)	HR (95% CI)*	HR (95% CI)^†^	HR (95% CI)^‡^
Age (years)											
<40	3.37	131	131	3331	590	2	0.6 (0.1–2.0)	1.0 (ref)	1.0 (ref)	1.0 (ref)	1.0 (ref)
40–50	3.38	137	140	2661	480	4	1.5 (0.5–3.6)	2.5 (0.5–13.7)	2.5 (0.5–13.8)	2.4 (0.4–13.2)	2.4 (0.4–13.2)
50–60	3.55	141	153	2873	558	23	7.7 (5.0–11.4)	12.9 (3.0–54.6)	12.2 (2.9–51.8)	12.4 (2.9–52.7)	11.9 (2.8–50.7)
>60	3.76	156	174	2159	434	23	10.6 (6.9–15.7)	17.6 (4.2–78.8)	14.7 (3.5–62.5)	15.8 (3.7–67.1)	15.2 (3.6–65.0)
Sex											
Female	3.56	139	144	6439	1142	17	2.6 (1.6–4.1)	1.0 (ref)	1.0 (ref)	1.0 (ref)	1.0 (ref)
Male	3.43	141	154	4686	920	35	7.5 (5.3–10.3)	2.8 (1.6–5.0)	3.0 (1.7–5.4)	2.8 (1.6–5.1)	2.7 (1.5–4.8)
BMI, body mass index (kg/m2)											
18.5–25	3.37	136	143	3925	718	13	3.3 (1.8–5.5)	1.0 (ref)	1.0 (ref)	1.0 (ref)	1.0 (ref)
≥25	3.57	142	151	6638	1233	35	5.3 (3.7–7.3)	1.6 (0.8–3.0)	1.4 (0.7–2.6)	1.5 (0.8–2.9)	1.5 (0.8–2.9)
Malignancy <5 years											
No	3.49	139	147	10594	1943	46	4.3 (3.2–5.7)	1.0 (ref)	1.0 (ref)	1.0 (ref)	1.0 (ref)
Yes	3.80	155	176	466	106	5	10.7 (3.9–23.8)	2.4 (1.0–6.1)	2.2 (0.9–5.6)	2.3 (0.9–5.9)	2.3 (0.9–5.8)
Smoking											
Never	3.48	143	142	3885	699	7	1.8 (0.8–3.6)	1.0 (ref)	1.0 (ref)	1.0 (ref)	1.0 (ref)
Ever	3.51	138	151	6981	1310	44	6.3 (4.6–8.4)	3.5 (1.6–7.8)	3.6 (1.6–8.0)	3.7 (1.6–8.1)	3.3 (1.5–7.4)
Major illness											
Never	3.49	139	147	10687	1972	47	4.4 (3.3–5.8)	1.0 (ref)	1.0 (ref)	1.0 (ref)	1.0 (ref)
Ever	3.80	158	181	437	90	5	11.4 (4.2–25.4)	2.6 (1.0–6.4)	2.0 (0.8–5.1)	2.2 (0.9–5.6)	1.8 (0.7–5.0)
Total cholesterol (mmol/L)											
≤6.2	3.47	137	145	8074	1491	27	3.3 (2.2–4.8)	1.0 (ref)	1.0 (ref)	1.0 (ref)	1.0 (ref)
>6.2	3.59	148	158	3042	569	25	8.2 (5.4–12.0)	2.5 (1.4–4.2)	2.3 (1.3–4.0)	2.3 (1.3–3.9)	2.3 (1.3–3.9)
HDL (mmol/L)											
>1.55	3.44	138	142	2824	507	9	3.2 (1.6–5.8)	1.0 (ref)	1.0 (ref)	1.0 (ref)	1.0 (ref)
≤1.55	3.52	141	150	8293	1553	43	5.2 (3.8–6.9)	1.6 (0.8–3.3)	1.6 (0.8–3.2)	1.6 (0.8–3.3)	1.5 (0.8–3.2)
Triglycerides (mmol/L)											
≤2.26	3.47	138	144	9506	1737	34	3.6 (2.5–4.9)	1.0 (ref)	1.0 (ref)	1.0 (ref)	1.0 (ref)
>2.26	3.67	153	169	1611	323	18	11.1 (6.8–17.3)	3.1 (1.8–5.5)	2.7 (1.5–4.8)	2.8 (1.6–5.1)	2.7 (1.5–4.9)
LDL (mmol/L)											
<4.9	3.47	138	145	9085	1686	35	3.9 (2.7–5.3)	1.0 (ref)	1.0 (ref)	1.0 (ref)	1.0 (ref)
≥4.9	3.67	149	161	2032	374	17	8.4 (5.0–13.1)	2.2 (1.2–3.9)	1.9 (1.1–3.5)	2.0 (1.1–3.6)	2.0 (1.1–3.5)
Kidney function, eGFR (ml/min)											
≥60	3.48	139	146	10540	1938	48	4.6 (3.4–6.0)	1.0 (ref)	1.0 (ref)	1.0 (ref)	1.0 (ref)
<60	4.18	183	211	213	49	3	14.1 (3.6–38.3)	3.0 (0.9–9.7)	2.3 (0.7–7.4)	2.6 (0.8–8.4)	2.4 (0.8–7.9)
Fasting glucose level (mmol/L)											
Normal, <5.6	3.47	137	145	9307	1712	35	3.8 (2.7–5.2)	1.0 (ref)	1.0 (ref)	1.0 (ref)	1.0 (ref)
Impaired, 5.6‐6.9	3.62	151	163	1379	260	9	6.5 (3.2–12.0)	1.7 (0.8–3.6)	1.6 (0.7–3.3)	1.7 (0.8–3.4)	1.6 (0.8–3.3)
Diabetes,≥7.0	3.73	169	173	431	88	8	18.6 (8.6–35.3)	4.9 (2.3–10.6)	3.9 (1.8–8.6)	4.1 (1.9–9.1)	4.4 (2.0–9.5)

*Note*: Mean factor levels in g/L for fibrinogen and IU/dl for FVIII and vWF.

Abbreviations: IR, incidence rate per 1000 patient years; HR, hazard ratio; 95% CI, 95% confidence interval; Obs yrs, observation years; CVD, cardiovascular disease events; HDL, high‐density lipoprotein; LDL, low‐density lipoprotein; eGFR, estimated glomerular filtration rate.

* Adjusted for fibrinogen level.

** Data on risk factors were missing for some cases.

^†^ Adjusted for Factor VIII level.

^‡^ Adjusted for von Willebrand factor level.

### 3.5 Analysis 3b. Effect of procoagulant factors on the association found in Analysis 3a

To explore whether procoagulant factors could mediate the association between shared risk factors and CVD events, we adjusted the observed HRs for fibrinogen, FVIII, and vWF levels (Table 4). This attenuated the risk estimates for the associations between increasing age, major illness, raised total cholesterol, raised TG, raised LDL, impaired kidney function and impaired and impaired fasting glucose levels and CVD events slightly, with fibrinogen associated with the strongest‐ and FVIII with the weakest attenuation of the HRs.

## 4 DISCUSSION

In this follow‐up study, we investigated whether the relationship between shared risk factors (for VT and CVD), and subsequent CVD was mediated through increased levels of procoagulant factors in patients with prior VT. In our first analysis, we showed that the presence of shared risk factors such as increasing age and BMI, dyslipidemia, the presence of a malignancy and major illness were associated with increased procoagulant levels. Our second analysis showed that increased levels of procoagulant factors were in turn associated with increased risk of CVD events. Finally, in our third analysis, we showed that the association between shared risk factors and cardiovascular events was partly attenuated upon adjustment for the procoagulant factors. This suggests that part of the relationship between shared risk factors and CVD in patients with prior VT may be explained by increased procoagulant factor levels.

Of the shared VT and CVD risk factors, age, the presence of high glucose levels, major illnesses, and impaired kidney function were associated with increased levels of procoagulant factors. These results are consistent with previous reports [17, 24, 25, 26, 27]. In our study, increased fibrinogen levels were associated with an increased risk of CVD, which is also in line with previous investigations [28, 29, 30]. It is estimated that 30%–50% of the interindividual variation in fibrinogen levels is heritable [31, 32], suggesting that at least part of the association we observed between higher levels of fibrinogen and CVD is of genetic origin. However, one must interpret this suggestion with caution since a meta‐analysis of genome‐wide association studies failed to show a causal association between genetically elevated fibrinogen levels (single nuclear polymorphisms; SNPs) and CVD events [33]. As opposed to fibrinogen, we detected weaker associations for elevated levels of vWF and FVIII with risk of CVD events. This is consistent with several large prospective studies on this issue. Smith and colleagues reported no increase in CVD risk in patients with increased FVIII or vWF levels as confidence intervals crossed unity [11]. In a case‐control study and meta‐analyses of 15 studies by Willeit and colleagues, the reported adjusted odds ratio for coronary heart disease was 1.16 (95% CI 1.10–1.22) for every increase of one standard deviation from baseline of vWF levels [34]. A cohort study among 1.5 million healthy blood donors showed that CVD events were slightly more common (i.e., a relative increase of 10%) in individuals with non‐O blood groups (who have higher levels of vWF and FVIII), as compared with those in blood group O carriers [35]. Our results are in line with these studies, as FVIII and vWF levels are approximately 25 IU/dl higher in blood group non‐O carriers [36, 37].

As expected, the presence of shared risk factors increased the risk of CVD events. We did a mediation analysis, adjusting the HRs for shared risk factors and CVD events for fibrinogen, FVIII and vWF levels. By doing so, it was possible to observe whether the increased risk could be explained by higher levels of these procoagulant factors. If the increased risk of CVD events would have been mediated through these procoagulant factors, that is, if they are part of the causal pathway, adjustment of these procoagulant factors should attenuate the observed HRs to unity. The results showed that there was indeed some attenuation of the HR after these adjustments, indicating that the studied procoagulant factors indeed may be in the causal pathway between the risk factors and actual occurrence of cardiovascular events. A possible explanation for our findings is that the risk factors contribute to an inflammatory state and/or endothelial damage, which leads to a rise in procoagulant factor levels [38, 39].

As far as we know, this is the first study demonstrating a relationship between various shared VT and cardiovascular risk factors, and procoagulant factors in patients with a previous VT. However, as this is the first report, and results are based on small numbers, it should be interpreted with caution. Since this study could only take few CVD events into account (*n* = 52), we were not able to analyze subgroups of ischemic stroke, myocardial infarction and peripheral vascular disease or investigate differences between diagnoses and procedures. Hence, the results of this study require validation in larger studies, preferably separately for separate ischemic stroke, myocardial infarction and peripheral artery disease. Another limitation is that the shared risk factors were measured at time of VT (index date), whereas blood was drawn (i.e., the start of follow‐up) several months later in time. The risk factors status of participants may therefore have been slightly different at the start of follow‐up than was actually measured at time of VT diagnosis. Furthermore, potential endothelial damage arising from the presence of shared VT and CVD risk factors leading to increased procoagulant factor levels may occur nearer to the event thereby resulting in a possible dilution of the effects we found. Unfortunately, we were not able to study this hypothesis due to the small number of CVD events. Finally, data on blood pressure or presence of hypertension were not available. Although hypertension is an undisputed major CVD risk factor, it is a contested risk factor for VT and may not classify as a shared risk factor [40]. The same applies to diabetes and dyslipidemia: although these are contested risk factors for VT [40], they increase the risk of CVD which is a reason why they were added to our analyses.

Our findings could be of clinical importance but need further clarification. Elevated procoagulant factors may be causally related to CVD and with shared risk factors. In turn, they may act as markers for underlying inflammation and endothelial dysfunction [41]. Managing cardiovascular risk factors by changing patient lifestyle and prescribing cardioprotective drugs such as statins could be appealing in the management of patients with VT to reduce both VT and cardiovascular risk. A potential use of procoagulant factors is to use levels, or rather the reduction of their levels to gauge the effectiveness of such management. It is interesting to note that in a randomized controlled trial including 247 patients with a VT, use of rosuvastatin resulted in improved coagulation profiles [42].

In summary, our findings support the hypothesis that increased levels of procoagulant factors may explain (part of) the increased risk of subsequent CVD in patients with previous VT.

## AUTHOR CONTRIBUTIONS

LJJS, EY, and WML contributed to the design of the study, analysis and interpretation of the data and wrote the manuscript. JFT was involved in the MEGA follow‐up study and critically revised the manuscript. FRR designed and performed the MEGA case‐control and follow‐up study and critically revised the manuscript. SCC contributed to the design of the study, interpretation of the data and critically revised the manuscript. All authors approved the final version of the manuscript.

## CONFLICT OF INTEREST

The authors declare that there is no conflict of interest that could be perceived as prejudicing the impartiality of the research reported.

## Supporting information

Figure S1. Flow chart of the selection of the study population

## Data Availability

The data that support the findings of this study are available from the corresponding author upon reasonable request.
