# Supplementary figures and images for: Procoagulant factors and future risk of arterial cardiovascular disease in patients with prior venous thrombosis: A cohort study
Source: EJHaem. 2023 Jan 17;4(1):3–12. doi: 10.1002/jha2.618 (PMC9928659; doi:10.1002/jha2.618)

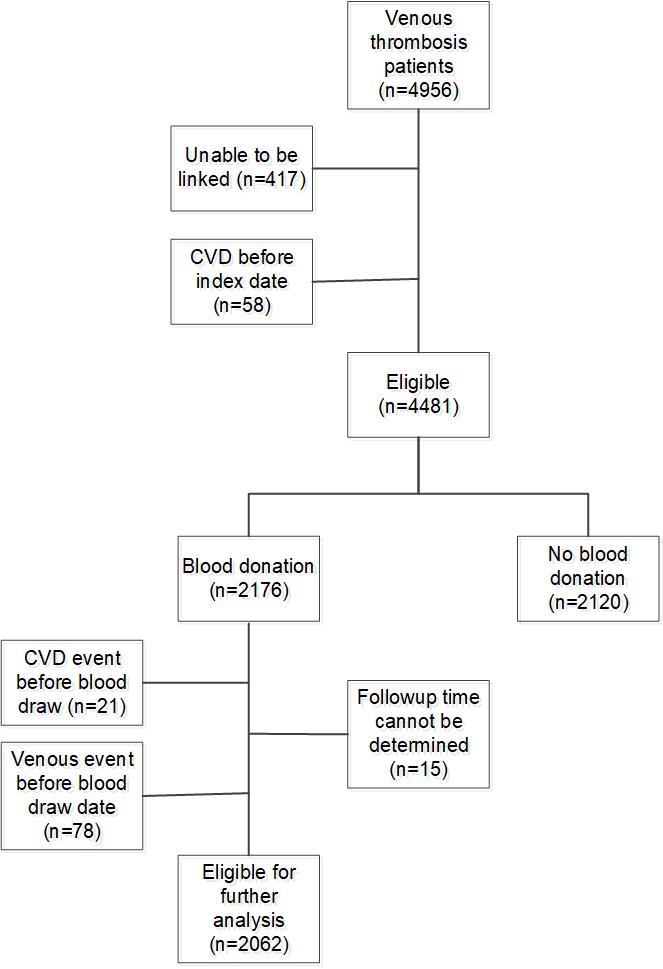


Supplementary Figure 2. Flow chart of the selection of the study population

Supplement: Supplementary file 1 — Figure S1. Flow chart of the selection of the study population [file JHA2-4-3-s001.docx]
